# “Peruvian balsam”: an example of transoceanic transfer of medicinal knowledge

**DOI:** 10.1186/s13002-020-00407-y

**Published:** 2020-11-09

**Authors:** Angela Schottenhammer

**Affiliations:** 1 History Department, Faculty of Arts, KU Leuven, Blijde Inkomststraat 21 – bus, 3307, 3000 Leuven, Belgium; 2 School of Economics, Shanghai University (SHU), Shanghai Baoshanqu, Nanchenlu 333, Building no. 3, rm 307B, Shanghai, 200444 China

**Keywords:** Peruvian balsam, Medicinal plants, Drug trade, Global history of medicine, Maritime history, Colonial Spanish America, China, Japan

## Abstract

**Background:**

Connections between China and the new Spanish colonies in America are known for an exchange of silver for silks and porcelains. That also medicinal drugs and medicinal knowledge crossed the Pacific Ocean is hardly known or discussed. *Myroxylon balsamum* (L.) Harms var. *pereirae* (Royle) Harms (“New World“ or “Peruvian balsam“) is a botanical balsam that has a long history of medicinal use, particularly as antiseptic and for wound healing. Except for a Chinese article discussing the reception of balsam in China and Japan, no scientific studies on its impact in China and Japan and the channels of transfer from the Americas to Asia exist.

**Methods:**

Description: (1) This section provides a general introduction into *Commiphora gileadensis* (“Old World” balsam) as a medicinal category and discusses the specific medicinal properties of *Myroxylon balsamum* (L.) Harms var. *pereirae* (Royle) Harms. The section “Historical research and uses” provides a brief survey on some historical analyses of balsam. Aim, design, setting: (2) Applying a comparative textual and archaeological analysis the article critically examines Chinese and Japanese sources (texts, maps) to show (i) what Chinese and Japanese scholars knew about balsam, (ii) where and how it was used, and (iii) to identify reasons why the “digestion” of knowledge on balsam as a medicinal developed so differently in China and Japan.

**Results and discussion:**

This chapter discusses the introduction of “Peruvian balsam” into, its uses as a medicinal as well as its scholarly reception in early modern China and Japan and introduces the channels of transmission from Spanish America to Asia. It is shown that *Myroxylon balsamum* (L.) Harms var. *pereirae* (Royle) Harms was partly a highly valued substance imported from the Americas into China and Japan. But the history of the reception of medicinal knowledge on Peruvian balsam was significantly different in China and Japan.

**Conclusions:**

In Japan, the knowledge on *Myroxylon balsamum* was continuously updated, especially through mediation of Dutch physicians; Japanese scholars, doctors and pharmacists possessed a solid knowledge on this balsam, its origin and its medicinal uses. In China, on the contrary, there was no further “digestion” or development of the knowledge on either *Myroxylon balsamum* (L.) Harms var. *pereirae* (Royle) Harms or *Commiphora gileadensis*. By the late nineteenth century, related medicinal and even geographic knowledge had mostly been lost. The interest in “balsam” in late Qing scholarship was pure encyclopaedic and philosophic.

## Background

**Fig. 1:**
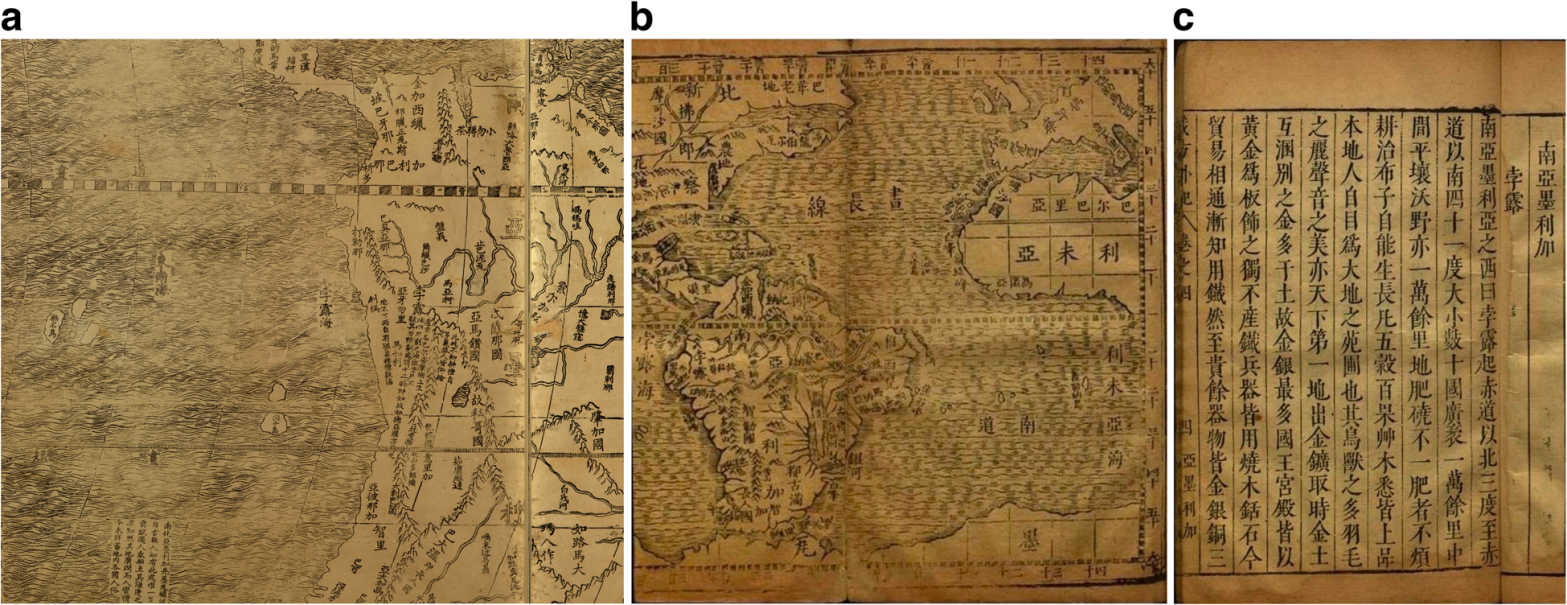
**a** Peru section from Matteo Ricci’s Kunyu Wanguo quantu 坤輿萬國全圖; **b** Map Peru section from Giulio Aleni’s Zhifang waiji 職方外紀; **c** Peru section from Giulio Aleni’s Zhifang waiji 職方外紀

Chinese scholars of the early seventeenth century knew that the region of Peru in Spanish America was rich in silver ore. In 1602, the world map *Kunyu Wanguo quantu* 坤輿萬國全圖 (Map of the Myriad Countries of the World; 1602; Fig. 1a) of the Italian Jesuit missionary, Matteo Ricci (1552–1610), had been printed. Ricci had prepared it for China’s Wanli Emperor (r. 1572–1620). With this map, Ricci introduced Western geographical knowledge, including information about the new Spanish colonies in America to the Chinese. The map had a great impact in China; it was published in five successive editions, attracting the attention of many scholars. Of particular interest is a section on Peru (Bolu 孛露), see Fig. 1a. Some 20 years later, Giulio Aleni (1582*–*1649), following Ricci’s format, drew his own map *Wanguo quantu* 萬國全圖, included in his geographical treatise *Zhifang waiji* 職方外紀 (Record of the Countries Beyond Imperial Administrations; 1623). Aleni also mentions Peru (Bolu 孛露), and speaks of “extremely rich gold and silver” ores (*gu jinyin zuiduo* 故金銀最多) [1]. Under the entry “Jinjiaxila 金加西蠟”, referring to “Castilia Del’oro”, that is, the new Spanish colonies in Latin America, silver and gold reserves are mentioned that “rank first under Heaven” (*tianxia cheng shou* 天下稱首), and “at the foot of the mountain there is a city, called ‘Silver City’” (Yincheng 銀城) [2]. This is definitely a reference to Potosí, rendered on his map as Boduoxi 波多西 Mountain, but not described in the text, [3] see Fig. 1b, c. Rumours even had it that a certain Mount Jiyi 機易山 (also 加溢, 交逸 or 佳逸), probably Cavite (?), the Spanish harbour on Luzon, would produce huge amounts of gold and silver [4]. The military official Yan Yinglong 閻應龍, and a local merchant called Zhang Yi 張嶷, memorialized the emperor that a certain Mount Jiyi “produces gold and silver, and by proceeding there by ship to wash for it, it will be possible to obtain 100,000 *liang* of gold and 300,000 *liang* of silver every year” [5]. Also *Dongxiyang kao* speaks of gold and silver as local products [6]. Interestingly, as we will see below, in addition to the references to silver, the just mentioned maps also speak of “balsam”. Why should balsam be mentioned on maps portraying the East Asian maritime world?

This brings us to the question of what else was known in China and Japan about Spanish America, and which products were, for example, imported into China other than silver? Medicinals were some of those other products, and medicinal knowledge definitely belonged to the kind of information that sometimes crossed oceans, perhaps unintentionally or even accidentally but that could revolutionize health systems in other countries. Balsams belonged to this kind of medicinals. In this connection, the present article wants to look at one particular plant, the resin of which was used for medicinal purposes, and for the embalming of corpses—this was the so-called “Peruvian balsam”, a botanical balsam that has a long history of medicinal use, particularly as an antiseptic and for wound healing. Except for two Chinese articles discussing the reception of balsam in China and Japan [7], basically no scientific studies on its impact in China and Japan and the channels of transfer from the Americas to Asia exist.

### Historical context and setting: balsam as a class of medicinal drug

Old World balsam was the most expensive aromatic plant in the ancient world. During Hellenistic and Roman-Byzantine times, *Opobalsamum* [that is, “juice of the balsam”] plant, or the “Balm of Judea” was frequently mentioned [8]. It grew wild in the Arab Peninsula but was rare. Only in Judea, and in the oases of Jericho and Ein Gedi was it cultivated, as Pliny the Elder (23–79 CE) noted [9]. Growing balsam was a monopoly of the ruler, and balsam sold for high prices. Originally cultivated under Jewish rule, the practice was continued under the Roman Empire, creating substantial revenue for the Roman treasury [10]. According to Pliny, pharmaceutical industries used every part of the plant: the wood (*xylobalsamum*), the fruit, the bark and the pure sap [11]. In the Roman middle period, cultivation moved to Egypt. As Zohar Amar and David Iluz emphasize, the balsam of the classical sources (*Commiphora gileadensis*) belongs to the incense plant family Burseraceae, including within it plants that still grow wild today in the dry, stony hills around the Red Sea [12]. While the collection of sap from frankincense and myrrh normally took many weeks to several months, the sap from the balsam tree, which was harvested at the same time, only needed an incision made with a knife, harvesting occurring relatively quickly [12].

Bertold Laufer provides a detailed discussion of the properties and uses of this “balm of gilead”, along with a physical description of the plant as described in ancient and middle period texts, as well as information on the tree’s distribution. “Balm of gilead” grew above all in the neighbourhood of Mecca and Medina, and in Abyssinia. It was transplanted to Palestine in historical times, where it was cultivated [13].

Carrying a unique aroma, “balm of gilead” was a valuable perfume among the social elites of the time, but was also renowned for its medicinal properties. To mention are its effectiveness against headaches, against early-stage cataracts and blurred vision. “Balm of gilead” was also used as a diuretic, as a curative for respiratory diseases and coughing and as an anti-toxin—for example, as a snake venom antidote [14]. In addition, it was used against cervical infections and in delaying menstruation [15], but, above all, for treating injuries and healing tissue [10]—an ability that balsam was also highly appreciated for in early modern times, as will be seen below. Balsam was, consequently, a renowned plant, similar to myrrh or frankincense, substances also highly valued in China since the early middle period.

Around 23 liters of resin sap could be produced per tree per year. In order to increase quantities, the sap was often mixed with a neutral carrier oil and other ingredients. As we will see, imported Peruvian balsam was also frequently used and traded as oil for this purpose. Balsam fetching high prices, it should surprise us little that various adulterations were also circulating. Pliny already stressed methods for detecting adulterations of the pure sap, namely involving its dilutability: balsam solidified in hot water and sank to the bottom of the container while the adulterated substance floated on top of the water like oil [16].

Balsam was traded across the Mediterranean and Indian Ocean worlds, and reached places as distant as China, where we find it transcribed in Tang times (618–906) as “*aboshen*” 阿勃參 (Table 1)—perhaps deriving from the Syriac word “*āpūrsamā*” (?) [17]. It reached China via the traditional overland Silk Roads but most probably also via maritime routes [18], shipped by Iranian and later also Arab merchants—Iranians and Islamized Huihui Arabs had migrated both into China’s northwestern but also into coastal regions where we find foreign Muslim settlements as early as late Tang times [19]. *Aboshen* is, for example, mentioned in the *Youyang zazu* 酉陽雜俎 (863) (Miscellaneous of the Youyang Mountain [in Sichuan]) by Duan Chengshi 段成式 (801 or 802–863) as a product of the Byzantine Empire (Fulin guo 拂林國) [20]. The entry is placed directly after the description of the myrrh tree (*moshu* 沒樹), *Commiphora myrrha*, which was important as incense and has been imported into China since antiquity:

**Table 1:** Chinese and Japanese plant and place names

Latin or English	Chinese	Japanese	Popular names
*Commiphora gileadensis*	*aboshen* 阿勃參, *Rudeya guo aboshen* 如德亞國阿勃參	阿勃參	(Old World) balsam; balsam of Judaea, Mecca balsam
*Myroxylon balsamum* (L.) Harms var. *pereirae* (Royle) Harms	*ba’ershamo/ba’ersamu* 巴尔娑摩, 拔爾撒摩, 拔爾撒摩, 巴爾撒木, 巴爾撒末, 拔爾撒彌, 巴拉薩嗎	巴尔婆摩, 巴尔娑摩, 拔尔撒摩, 拔律殺沒, 拔爾撒謨, *balsamo* バルサモ	(New World) balsam, Peruvian balsam, balsam of Peru
*Cinchona officinalis*	*jinji’na* 金鷄納	金鷄納	Jesuits’ bark; *árbol de calenturas*
This term refers to several plants belonging to the genus *Smilax*	*tu fuling* 土伏苓	土伏苓	China root
Bolivia			
Brazil	Boleixili 伯肋西理, Boxi’er 伯西爾, Bahia 巴以亞 (?)		
Byzantine Empire	Fulin guo 扶林國		
Castilia	Jinjiaxila 金加西蠟		
Charcas		Charkasha 察尔加沙	
Concho	Ganshu 乾庶		
Juríes (?)		Lushi 魯私	
Lima	Lima 利禡, 里馬	利禡	
Mount Jiyi (Cavite?)	Jiyishan 機易山, 加溢, 交逸, 佳逸		
Peru	Bolu 孛露, Bilu 畢錄, 壁露, 秘魯, Bailu 白露	孛露, 百露, Peru ペル, Peruvian 瞥律匪坑	
Potosí	Boduoxi 波多西, Yincheng 銀城		
Quito		Qido 祁多	

*This list contains the plant and place names as they appear in the analysed historical maps and texts

*“Aboshen* originates from the Byzantine country. It is more than 1 *zhang* high [*ca*. 3.33 m]. Its bark has a greenish-whitish colour. The leaves are fine and always two face one another. The flowers are upright like rape turnips [*Brassica rapa* var. *rapa*], and the buds are reddish like the colour of ripe black pepper (*hujiao* 胡椒). Cutting its branches, a sap [leaks] like oil. Taken against scabies and sores, there is no disease it cannot heal. Its oil is very valuable and is as expensive as gold.”

阿勃參出拂林國長一丈餘皮青白色葉細兩兩相對花似蔓菁正黄子似胡椒赤色斫其枝汁如油以塗疥癬無不瘥者其油極貴價重於金.

This description attests to the fact that already Duan Chengshi knew about the excellent properties of balsam against skin diseases. And he, too, mentions the high price of balsam.

Later works copied the entry by Duan Chengshi on *aboshen* [21]. Balsam was particularly used in Muslim medicine, which was introduced into China after the Tang period, especially since the thirteenth century during Yuan times (1271–1367) [22]. Balsam appears as a remedy various times in the late Yuan, early Ming period *Huihui yaofang* 回回藥方 (Muslim Medicinal Recipes) [23]. We find, for example, the following entries:

[Ar.] *Ḥabb l-balasān* [balm of Gilead, *Commiphora opobalsamum*; *Sambucus nigra*] ([subtext] this is a pill medicinal made from an oil from a tree in the [al-]Misr [Egypt] land) *…*

[Pr.] *‘Ud-e balasān* [wood of *C. opobalsamum*] ([subtext] this is a black-colored myrrh) *…*

Use oil of [Ar.] *balasān* [balm of Gilead, *Commiphora opobalsamum*] ([subtext] [Pr.] *roughan-e balasān* [“oil of balm”]) ([subtext] *roughan-e balasān*) to smear the hand. *…*

[Pr.] *Balasān* [balsam-tree, balm of Gilead, *Commiphora opobalsamum*] seeds ([subtext] *tukhm-e balasān* [“balsam-tree seed”]) [24].

As we will see below, the expression *aboshen* appears in the sources until Ming times, and the Chinese knew balsam as a product originating from the eastern Mediterranean and the Arab worlds. And also the medicinal qualities of balsam, especially against skin diseases and sores, were already known in China during Tang times. It was only with the advent of the Jesuits that balsam from the New World, that is, from the new Spanish colonies in America, was introduced—the balsam of Peru.

Actually, the balsam of Peru—that belongs to a quite different family, Fabaceae, that is interestingly nowhere close to the family of the Old World balsam, Burseraceae—originally came from the part of Central America known as Salvador or balsam coast, so the name is at least misleading. Albert Hale traces this misnomer back to European ignorance of the American continent. Next to Mexico, Peru was the land of El Dorado and everything originating from the Pacific “was apt to be called Peruvian, whether from Peru itself, from Chile, or the mysterious Potosí” [25]. I would argue though that the misnomer has to be traced back to the fact that the balsam—as well as many other products too—was originally shipped from the port of Callao, Lima, in the Viceroyalty of Peru. The so-called “balsam coast” extends along the western Pacific coast of Salvador between the ports of Ajacutla and La Libertad, just approximately 40 miles of coast. The tree grows in the coastal regions of modern El Salvador, Nicaragua, Honduras, Guatemala, Mexico, Costa Rica, Panama and Cuba at approximately 300 to 700 m above sea level.

Albert Hale mentions that Peruvian balsam has been shipped in coconuts and, consequently the shipped product was also called “cocoa balsam”. The ancient native name for it was “*hoitziloxitl*” [26]. The outer wood of the balsam is white, the inner part red or even black, and due to its extraordinary hardness, Peruvian balsam wood is also good for furniture or even construction.

Although the Peruvian balsam trees produce sap the entire year, the sap is most abundant in the dry season, in summer. In the early twentieth century, when Albert Hale was writing, there were balsam gatherers living close to the trees. They collected and sold the sap to merchants for further distribution [27]. He describes the process of recovering the sap as follows: the gatherers make rectangular holes in the bark of the tree, between 25 to 28 holes of 15 to 16 cm in depth. For 5 to 8 days, the sap flows slowly, but steadily. The natives use very clean cloth to collect it.

After 8 to 10 days of slow, steady flow, the flow of the balsam stops and has to be reinitiated by very carefully applying heat using burning torches. This kind of procedure is repeated year after year. As a rule, trees recovered fast, but there was a need for care. One had to pay attention to trying not to extract too much sap at any one time in order to prevent injuring or even destroying the tree.

Adulteration is done by mixing the pure sap with raw sugar, or with the husks of cacao diluted in water. When the collection process is over, the pieces of cloth are put into water and boiled for half an hour, impurities skimmed off. As the balsam is still in the meshes of the cloth, the cloth is put into a primitive press to squeeze out the balsam substance, so as to make it settle to the bottom of a kettle (the specific gravity of balsam is higher than of water).

Another purification process follows. What remains is the pure or crude balsam that is then sold and, as a rule, undergoes another clarification process to drive out water through evaporation. A healthy balsam tree would, thereby, provide between 3 and 4 lb of sap annually [28]. The historical use of balsam, Albert Hale, expounds, was almost entirely surgical. “Applied to wounds, balsam seemed to have a wonderful power to stimulate the healing process, while being at the same time a natural anti-septic, incapable of doing harm”—and it was still used like this in the early twentieth century, effective also in skin diseases and parasitic irritations. Cinnamic acid, one of its ingredients, has been considered helpful when applied, for example, against tuberculosis [29].

## Methods

The methods applied in this article are (1) a discussion of the general medicinal properties and uses of balsam, including a brief survey on historical studies on balsam; and (2) a critical comparative analysis of Chinese and Japanese textual and archaeological sources (especially texts and maps) to show (i) what Chinese and Japanese scholars knew about balsam, (ii) where and how it was used and (iii) to identify reasons why the “digestion” of knowledge on balsam as a medicinal developed so differently in China and Japan.

### Description: medicinal properties and uses

**Fig. 2:**
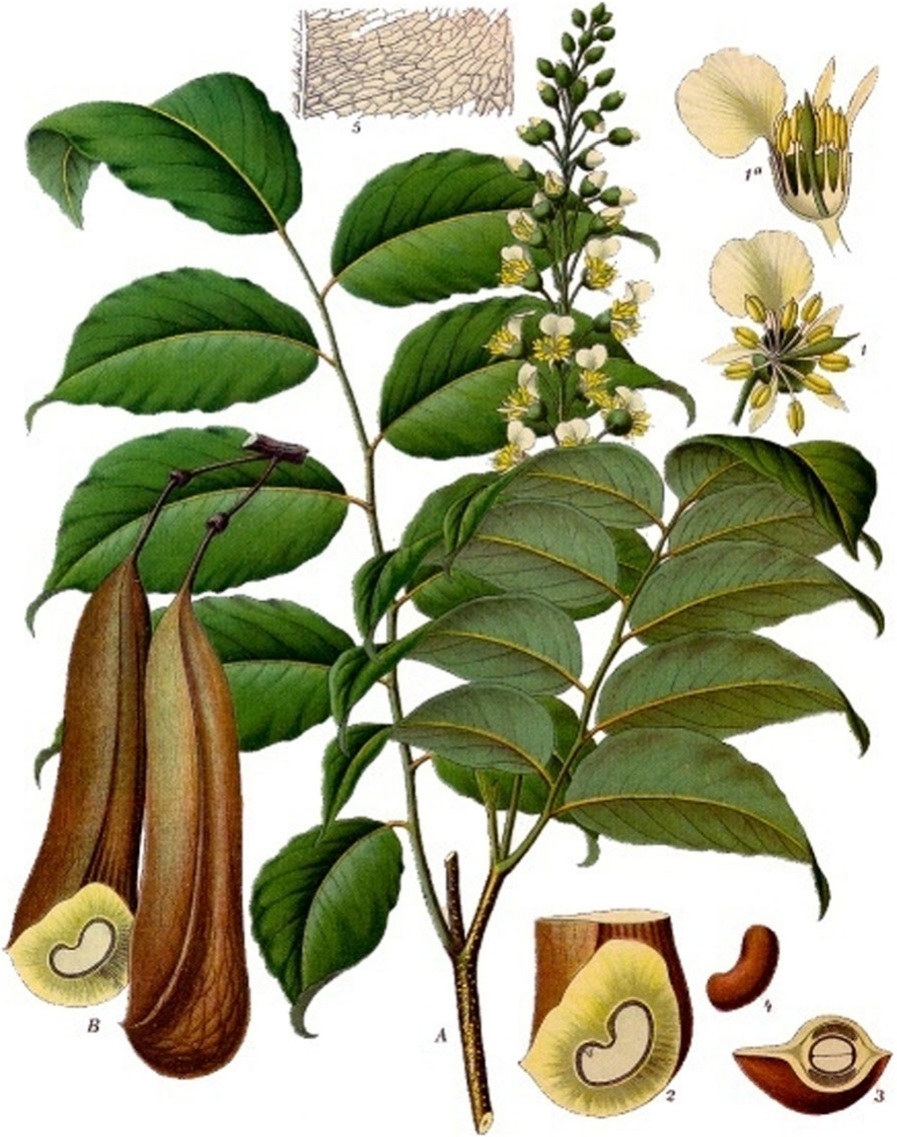
*Myroxylon balsamum* (L.) Harms var. *pereirae* (Royle) Harms

Balsam of Peru is a resin obtained from the bark of the tree *Myroxylon balsamum* (L.) Harms var. *pereirae* (Royle) Harms (Fig. 2) [30]. Apparently, little information is available on the qualitative and quantitative chemical composition of Peruvian balsam. The oleoresin is said to contain some 250 ingredients with a resin content of 20% to 40% [31]. Major components are benzyl cinnamate and Benzyl benzoate and it is still used today and approved by the Food and Drug Administration (FDA) [32]. It is approved and categorized today as a herbal medicine product [33]. Balsam of Peru increases blood flow to wound areas and also helps to fight bacteria, qualities that explain the excellent healing qualities that are ascribed to it in our historical sources. Today it is used, in combination with other medicinals, to treat sores and other skin ulcers.

Peruvian balsam, either black or white, was obviously very popular in the early modern world, although in seventeenth-century Europe also doubts about its efficiency arose [34]. The balsam constituted a definite component of many shipments from Spanish America to Asia. R. M. Pacheco Olivera has identified seven galleons departing from Mexico that carried Peruvian balm as part of the cargo, the *San Joseph* in 1772, *Nuestra Señora de la Lu*z in 1775, *San Joseph* in 1780, *San Andrés* departing from San Blas in 1786, the *San José* in 1788, one unidentified galleon in 1791, carrying also vinegar and the galleon *Nao Magallanes* in 1809 [35]. Balsam of Peru was, for example, used in wound treatments and chest ailments [36]. But we also know that other kinds of balsams, mostly as oils, were shipped from Spanish America to Asia, although the Spanish shipped large quantities to Spain as well, and the Spanish were, by far, not the only suppliers of medicinal plants from Spanish America [37]. As Stefanie Gänger stresses, “(t) hough the exact routes and the volume of illegal trade in America’s medicinal plants elude us—the difficulty of pursuing the plants onto a contrabandist’s vessel or through the bustle of a marketplace render any mapping or quantification necessarily fragmentary—the paper trail in Spain’s archives leaves little doubt that foreign merchants handled a significant volume of contraband, and that Spain faced ‘much difficulty’ in closing the trade’s many ‘gateways and entries’” [38].

By the early to mid-eighteenth century, Peruvian balsam, *cinchona* (*Cinchona officinalis*, Chin. *jinji’na* 金鷄納, also called “Jesuits’ bark”) [39], the bark of which was used for medicinal purposes [40], *sarsaparilla* [41] (very similar to the so-called “China root”, *tu fuling* 土伏苓 in Chinese) [42], that is, several plants belonging to the genus *Smilax* and used against the early modern scourge of syphilis, *jalap root*, whose turnip-shaped roots were used as purgative, *guaiacum* (*Guaiacum officinale* (LINN.) [43], a mild laxative and diuretic, and *ipecacuanha* (*Carapichea ipecacuanha*) [44], widely used as a syrup to cure diseases such as influenza, belonged to the basic stocks of pharmacies in Europe’s cities [45]. According to the 1748 report of the Jesuit Juan Francisco Toro from Lima, *cinchona*, *guaiacum*, *ipecacuanha*, but also *contrayerva*, a medicinal rhizome of various tropical Central American and South American species of *Dorstenia* of the family Moraceae that is also used to induce sweating or used against snakebites, and Peruvian balsam, were among the most commonly used remedies in the practice of medicine in Peru [46].

Peruvian balsam was also tested as a treatment against scurvy. It is mentioned by James Lind (1716–1794) among various other medicinal drugs used for this purpose [47]. The effects of balsam in preventing scurvy, however, turned out to be disappointing and could be discarded after experimenting. Sir Gilbert Blane (1749–1834), in a list of drugs frequently used in the British Royal navy, records a total of sixty-three medicinals, among them Peruvian bark and balsam, but used for wounds [48].

It is clear that there also existed a market for certain medicinals from Spanish America in Asia, definitely in China and Japan. As Stefanie Gänger stresses with reference to a letter to the Marques de Sonora (1720–1787) dated October 19, 1786, the Viceroy Antonio Caballero y Góngora repeatedly mentioned plans to increase the consumption of *cinchona* in Asia and export it via Acapulco to the Philippines [49]. Peruvian balsam and *cinchona* were also among the medicinals that were imported into Japan through the port of Nagasaki by merchants from various countries, including Portuguese and Dutch, active in the resale of these drugs [50]. These medicinals were available and traded further across the Indian Ocean or via the Atlantic to cities as far away as Istanbul and Smyrna (modern Izmir), also reaching markets in Syria or Iran [51].

Bernabé Cobo (1580–1657), who lived in Potosí, serving in the local Jesuit Mission there between 1615 and 1618, describes the Peruvian balsam as follows:

The tree which produces Balsam in these Indies is not of a single species, but of three or four. This liquor is very similar to that of the Syrian Balsam, and not inferior to it in aroma and virtues. One species of tree which yields this Balsam, and in the greatest quantity, grows in the diocese of Guatemala where I saw it, and in other warm lands. It grows larger than a mulberry, and has a thick trunk, and fragrant as well as tough wood … . On scarifying the trunk of this tree a liquid exudes which we call Balsam, the colour of treacle, a blackish red, of a sharp, somewhat bitter flavour and a strong but pleasing smell. This liquid is also extracted in another way, which is to boil in water the shoots and tender twigs especially picked for this purpose, immersing them in an earthenware vessel with nothing but water … [52]

Cobo speaks here of several species of balsam trees. The first one, which he claims to have personally seen, was indigenous to the San Salvador district of Guatemala, and it was the balsam, which, according to A. W. Haggis, commercially supplanted the “real” balsam from Peru, which was shipped across the Pacific from the port of Callao, Lima [53]. The real and much superior balsam of Peru was that which Cobo describes by the Quichuan name of “Quina-Quina”, indigenous to Peru.

This is consequently an early description of the balsam tree, botanically *Myroxylon balsamum* var. *pereirae*. That neither of these two described balsam trees was identical with the *genus Cinchona*, or the “Jesuits’ bark” from Peru, becomes very obvious when we read Cobo’s description of a so-called fever tree: “In the district of the city of Loja, diocese of Quito, grows a certain kind of large tree, which has bark like the cinnamon, a little more coarse, and very bitter; which, when ground to powder, is given to those who have a fever, and with only this remedy, it leaves them. Having taken a quantity of this powder, to the weight of two *reales*, in wine or in some other liquid, soon after it reduces the temperature. These powders are now very well-known and esteemed, not only in all the Indies but in Europe, and are urgently sent for and demanded from Rome” [53].

This description clearly refers to *Cinchona*. An even earlier text, printed in 1638 and written by an Augustinian monk, Antonio de la Calancha (1584–1684), also describes this same “fever tree”: “A tree grows which they call ‘the fever tree’ (*árbol de calenturas*) in the country of Loxa, whose bark, of the colour of cinnamon, made into powder amounting to the weight of two small silver coins and given as a beverage, cures the fevers and tertianas; it has produced miraculous results in Lima” [54]. I mention this here in this context because records on the balsam tree of Peru are sometimes confusing, so that one starts to ask which species or *genus* a description actually refers to [55]. And the bark of the tree that was commercially sold as a drug most probably did not always stem from the *cinchona* tree, but may well have come from the Peruvian balsam tree (or even other trees).

### Historical research and uses

Research on the balsam of Peru started in the seventeenth century in early modern Europe. In this context, I would like to mention, for example, Johann Adrian Slevogt's (1653–1726) dissertation (1705) *Balsamum verum quod vulgo opobalsamum dicitur* [56]. This botanical dissertation, specifically dedicated to the investigation of balsam, explains its characteristics, varieties, and uses. It includes several references (p. 18, 25) to the balsam of Peru. At the end of this work, Slevogt also includes his opinions on Peruvian bark (*cinchona*). There is also the dissertation of Johann Wilhelm, carried out under the supervision of the German physician and chemist, Frederick Hoffmann the younger (1660–1742), professor of medicine at the University of Halle [57]. The dissertation examines the use of turpentine oil as a medium for applying Peruvian balsam in the treatment of various diseases. Accordingly, it could be used against scabies, worms, kidney disease, gonorrhoea, and conditions of the digestive and urinary tracts.

The balsam tree—mostly used as an oil (that is, the resin of the balsam tree), fragrance or fragrance and incense balls (*xiangqiu* 香球)—and knowledge about its application and uses also entered China and Japan and became a highly valued substance among various members of society. It was especially used for medicinal purposes but also for preserving dead corpses. We also know that balsam was used as an ointing oil in churches and cathedrals: In 1756, for example, Juan de la Fuente Yepes, bishop of the cathedral Nueva Segovia in Manila, in a letter requested from the authorities in Mexico, among many other items, four ounces of an ointing oil for consecration (4 onzas de bálsamo para la consagración de los Santos Óleos) [58].

## Results

### Evidence from Chinese texts and maps

Under the entry Peru, the famous map by Matteo Ricci, *Kunyu Wanguo quantu* (see Fig. 1a), refers to a “fragrance” (*xiang* 香) called “*ba’ershamo*” 巴尔娑摩, a tree which produces an oil, that is a resin, when cut; “used to balsam corpses, they do not decompose”. And the point where the tree has been cut entirely recovers after 12 h (*zhou shi’er shi ji ru gu* 周十二時即如故). (The balsam) also occurs in Judaea (*Rudeyaguo yi you zhi* 如德亞國亦有之) [59]. This balsam of Judaea (also “Mecca balsam”) was used in the ancient and middle period Mediterranean area, as we have seen above. Ricci was consequently aware of the use and importance of balsam in the ancient world, and links it up to the balsam from the New World. This implies that both balsams possessed similar or even almost identical properties, or were at least used in similar ways.

Chen Ming draws our attention to the novel *Sanbao taijian Xiyangji tongsu yanyi* 三寶太監西洋記通俗演義, attributed to Luo Maodeng 羅懋登. Scene 86 mentions that the king of Aden presented Zheng He with balsam (阿勃參) as a gift. But the description of this balsam is obviously taken directly from Luo Yuejiong’s 羅曰褧 *Xianbin lu* 咸賓錄 (Records on All Guests), preface dated 1591, so that Chen Ming concludes that knowledge and use of balsam during the Ming period was very restricted [60]. *Xianbin lu* states that “balsam oil is very efficient as an ointment against scabies and sores, but its price is extremely high” (阿勃參油宜塗癬疥大效價极貴) [61]. The information that Zheng He received balsam as a gift is not included in the Aden sections of Ma Huan’s 馬歡 (*ca*. 1380–1460) *Yingyai shenglan* 瀛涯勝蘭 [62]. This does not, of course, prove that information from Luo Maodeng was incorrect. Against the background that the balsam was a very valuable product, and in fact originated from the local neighbourhood, it seems, on the contrary, quite possible that the king of Aden presented balsam as a special gift. And one would also wonder where Luo Maodeng should have gotten his information from, if this statement was pure fabrication.

The Ming period *Xiangcheng* 香乘 (Chariot of Aromatic Substances) by Zhou Jiashou 周嘉胄 (1582–1658) changes the entry “*zhi jiexuan*” 治疥癬 to “*zhi lai*” 治癩, specifying that the balsam is applied to cure skin diseases [63]. Shen Maoguan’s 慎懋官 (fl. 16^th^ cent.) *Huayi huamu niaoshou zhenwan kao * 華夷花木鳥獸珍玩考 (An Investigation of Plants, Animals and Rare Things of China and the Barbarians, 1581) introduces rare or exotic things from China and the rest of the world [64]. Here, we still encounter the balsam of the Middle East (阿勃參). Shen Maoguan says it originates from Fulin guo 扶林國 [65]. Finally, at the end of the Ming period, the terms 巴尔娑摩 and 拔爾撒摩 (“*ba’ershamo*”) appear. This means that most scholars of the mid to late Ming period still received and copied their information from older texts, and that the balsam of Peru was actually only introduced through European missionaries, and included into Chinese literary and medicinal texts by those authors who took note of the newly introduced Western knowledge.

Aleni speaks of “a tree, which produces a grease that is extremely fragrant and is called *ba’ersamo*; it is used to treat all kinds of injuries; within one night, the muscles and flesh are totally recuperated as before. Applied on smallpox, they do not leave scars, embalming dead corpses, they will not decompose in myriads of years”.

**Fig. 3:**
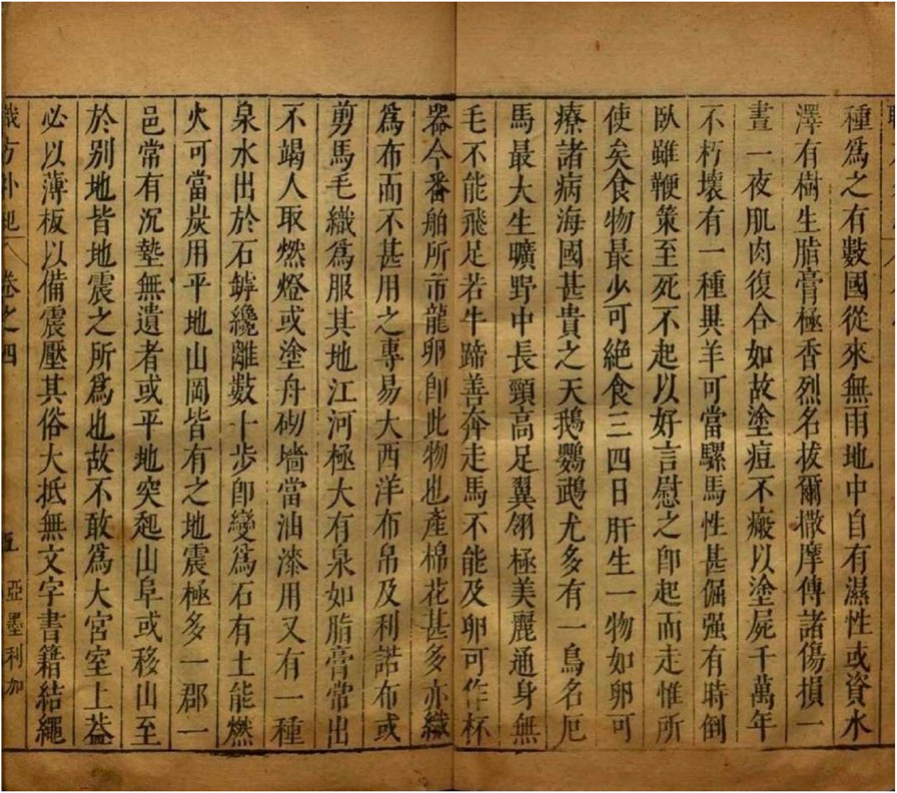
Entry on the “balsam of Peru” in the Peru section of Zhifang waiji 職方外紀

有樹生脂膏極香烈名拔爾撒摩傅諸傷損,一晝一夜肌肉復合如故,塗痘不瘢,以塗屍,千萬年不朽壞 [66] (Fig. 3).

The *Wuli xiaoshi* 物理小識 (Notes on the Principles of Things) by Fang Yizhi 方以智 (1611*–*1671) also introduces the “balsam of Peru”. Fang Yizhi aimed to comprehend the seminal forces of natural change and presented his opus as a collection of observations, findings and other sources. He was open-minded and generally accepted European explanations of natural phenomena, except when they resulted in religious explanations [67]. Most of his information when talking about the Peruvian balsam stems from the Peru-chapter in *Zhifang waiji*, which suggests that this work was circulating among scholars interested in knowledge brought to China by Jesuits and other Europeans. In Chapter 9, he adds that this balsam is intensely fragrant and that all wounds and injuries are healed (lit. “muscles and flesh are reunited”) within one day and one night; applied on smallpox, no scars on the skin will be left, applied to dead corpses, they will not decompose even after myriads of years (中通曰穆公云孛露有樹生脂膏極香烈名拔爾撒摩傅諸傷損一日夕肌肉復合塗痘孛不瘢以塗屍千年不朽壤) [68]. This information is clearly taken from Aleni’s Peru chapter.

The Flemish Jesuit missionary Ferdinand Verbiest (1623–1688) also mentions the Peruvian balsam in his 1674 *Kunyu tushuo* 坤輿圖說 (Illustrated Explanation of the World), basically copying the information from Aleni [69]. The world map of a certain Francesco Sambiasi (1582–1649; Chin. Bi Fangji 畢方濟), copies of which exist in the libraries of Ghent University and Turin, also draws on information from Matteo Ricci and Guilio Aleni and mentions the “balsam of Peru” [70].

During the Kangxi period, Fan Shouyi 樊守義 (1682–1753), a Chinese Christian, born in Shanxi, and who later became a Jesuit missionary [71], was one of the first Chinese known to have travelled to Europe. He later left behind his travel records, *Shenjian lu* 身見錄 (Record of Personally Seen Things) [72]. In his notes, he records that he left China through the port of Macao, and reached Bahia 巴以亞 (modern Brazil) from whence his journey took him further on to Lisbon. His notes also refer to a local “balsam fragrance, an oil [used to treat] sword and knife injuries” (巴爾撒木香刀傷油) [73]. That he does not speak of Peru as place of origin but of Bahia might indicate that this was the region or port from where the balsam was further shipped to Europe. The large early eighteenth-century encyclopaedia *Gujin tushu jicheng* 古今圖書集成 (Comprehensive Collection of Books and Pictures of the Past and Present, first published in 1726) mentions it in an entry on Peru 白露, in the section “Borders” 邊裔典 [74].

Chen Ming also introduces Zhao Xuemin’s 趙學敏 (1719–1805) *Bencao gangmu shiyi* 本草綱目拾遺 (Additions to the General Compendium on *Materia Medica*) that states: “Balsam ‘*Kunyu tushuo*’: name of a tree, originating from Peru. This tree produces an oil that is intensely fragrant, it can be used to make medicine. All wounds and injuries are healed (lit. “muscles and flesh are reunited”) within one day and one night; applied on smallpox, no scars on the skin will be left, applied to dead corpses, they will not decompose even after myriads of years” (拔爾撒摩《坤輿圖說》: 木名,出白露國,此樹生脂膏極香烈,可入藥。敷金刃傷,一晝夜肌肉復合如故,塗痘不瘢,塗屍千年不腐) [75].

The “balsam tree” is, interestingly even mentioned on an untitled 1743 Chinese map. A circle is shown in the maritime space southeast of China, and includes the text “White mountain peak(s) [i.e. the Andes mountains?]. In Peru, there is a balsam tree that produces an oil; when the tree is cut with a knife, the oil leaks out, applied on dead corpses, these do not decompose (孛露國有巴爾婆[=撒]摩樹上油以刀取之塗尸不敗) [76]. On the one hand, this information attests to the relative importance of Peruvian balsam in the work of some map drawers. On the other hand, this entry clearly shows that the use of the balsam for mummifying corpses was obviously considered more important and interesting than its use for medicinal purposes.

According to the *Da Qing huidian shili* 大清會典事例 (Collected Statutes of the Qing Dynasty: Precedents), in 1727, the Portuguese royal envoy Alexandre Metello de Souza e Menezes (1687–1766) presented the Yongzheng Emperor several gifts, among them “St. Thomas, Peruvian and Brazilian balsam oil” (聖多黙巴爾撒木油, 壁露巴爾撒木油, 伯肋西理巴爾撒木油) [77]. Also *DaQing yitong zhi* 大清一统志 (Records of the Unity of the Great Qing; 1790), an imperial geography and description of the Qing empire, mentions St. Thomas, Peruvian and Brazilian balsam as a product of the Europeans (lit. Western oceans) [78].

In the nineteenth century, Zhang Peiren 張培仁 (Jinshi 1847) records under “strange things heard from overseas” (海外異聞) the fact that “overseas there is a country called Bilu. It has a balsam tree that produces an extremely fragrant oil; it rapidly heals injuries as well as smallpox so that no scars remain; applied to dead corpses, they will not decompose” (海外秘魯國有拔爾撒摩樹生脂膏極香敷傷卽合敷痘不瘢敷尸不腐) [79]. That Zhang Peiren by the later nineteenth century considered this knowledge on Peru that had been copied and transported since approximately 1600 in Chinese literature and on maps, as strange and exotic, might suggest that knowledge about the background and the interest in this balsam had been lost by then.

**Fig. 4:**
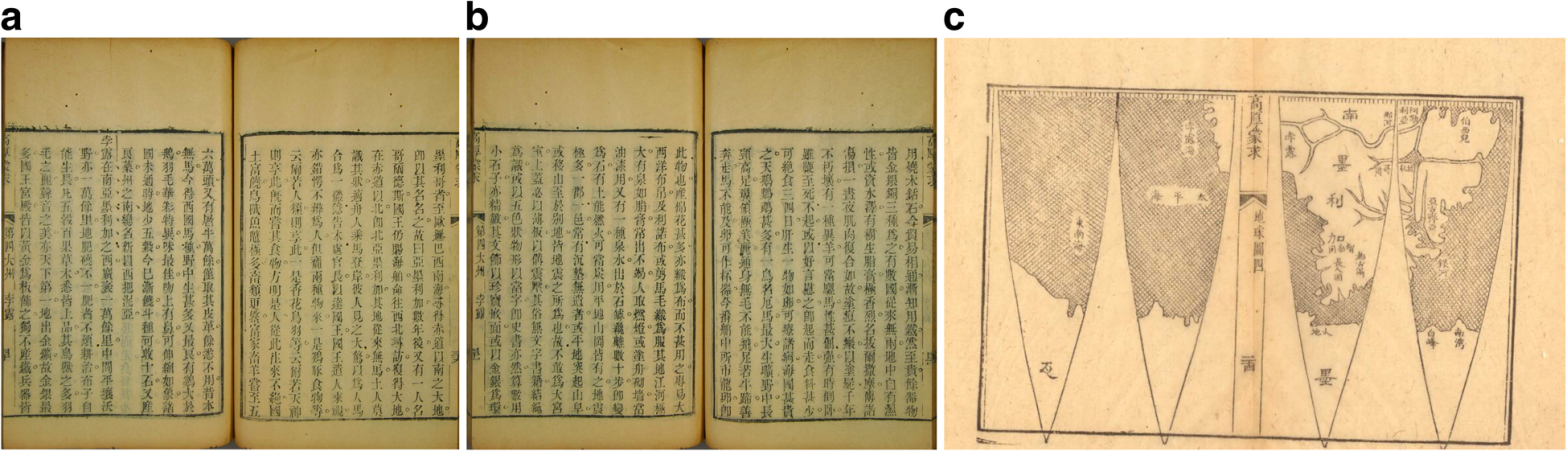
**a**) Peru section in Gaohou mengqiu 高厚蒙求 (p. 40a), **b**) Peru section in Gaohou mengqiu 高厚蒙求 (p. 40b), **c**) Map of Spanish America including Peru and the Pacific Ocean in Gaohou mengqiu 高厚蒙求

Around the same time, Liang Tingnan 梁廷楠 (1796–1861) in his *Haiguo sishuo* 海國四説 (Four Treatises on Maritime Countries; 1846), like *DaQing yitongzhi*, mentions Peruvian and Brazilian balsam as an Italian and Portuguese “tribute” gift (聖多黙巴爾撒木油, 壁露巴爾撒木油, 伯肋西理巴爾撒木油), used, for example, against a cold (*biyan* 鼻烟) [80]. A reference is included to *Gaohou mengqiu* 高厚蒙求 (*Tianxue rumen* 天學入門, A Study of the Universe for Beginners, 1815) by Xu Chaojun 徐朝俊, also entitled *Astronomy with Star Charts*, which has a section on regions overseas (海域大觀) and a longer paragraph on Peru 孛露, located in the west of South America (see Fig. 4a–c) [81]. Xu Chaojun also speaks about the rich silver ore, the flora and fauna and basically copies the information provided in *Zhifang waiji*. The reference to the balsam stands on page 40b [82].

Wei Yüan’s 魏源 (1794–1857) *Haiguo tuzhi* 海國圖志 (Illustrated Survey of Countries Beyond the Seas; 1844) says: “There is a tree that produces a grease (oil) which is extremely fragrant and is called balsam; it is used to treat all kinds of injuries, within one night, the muscles and flesh are totally recuperated as before. Applied on smallpox, they do not leave scars. Applied to dead corpses, these do not decompose even after ten thousands of years” (有樹生脂膏極香烈名拔爾撒彌傅諸傷損一晝一夜肌肉復合如故傅痘不瘢以塗尸千萬年不朽壞) [83]. Hu Tingguang 胡廷光 (active eighteenth century), a Chinese doctor who had specialized in treating traumatic injuries, also refers to the “balsam tree” and the oil used to treat wounds and injuries as products of Peru in his *Shangke huizuan* 傷科彙纂 (1815). He skips the information of its use for the embalming of corpses (白露國有樹生脂膏極香烈名爾撒摩傅傷損一晝夜肌肉復合如故) [84].

## Discussion

### Use of balsam as medicinal in China and Japan

#### China

Since approximately the mid-sixteenth century, European missionaries brought Peruvian balsam from Spanish America into Asia. Macao, founded by the Portuguese in China in 1557, played a major role in this as an international crossroads between East and West. In 1594, the Colégio de São Paulo (Shengbaolu xueyuan 聖保錄學院) was founded by Portuguese Jesuits as a first Western-style college in China. The college is often referred to as the cradle of Western sinology, and it played a significant role also in the transfer of medicinal and pharmaceutical knowledge from Europe to China. It also had its own pharmacy. Its collection of medicinal prescriptions, the “*Yesuhui bifang*” 耶穌會秘方 (Secret Recipes of the Jesuits), includes thirty-seven prescriptions containing mainly Western medicinals. Balsam (Port. “*balsamo*”) is frequently mentioned in these recipes [85]. A famous Mexican Franciscan missionary who came from the Colégio de São Paulo and later practiced in Changshu, Jiangsu, was Pedro de la Piñuela (1650*–*1704). From Macao and Guangdong, Peruvian balsam gradually spread along the Chinese coasts and also reached the Manchu Qing court in Beijing, mainly as a valuable gift and precious medicinal.

Xiao Shi’s 蕭奭 *Yongxian lu* 永憲錄, 1725 (Records from the Yongzheng Period) notes that, in 1722, a list of the Jiangxi commissioner, Wang Qijing 王企靖, included one bottle of balsam fragrance oil against cold [86]. *Qingdai Guangdong gongpin* 清代廣東貢品, among many other medicinals, such as a box with nutmegs, six jars of sandalwood oil, two bottles of clove oil or eugenol (aromatic oily liquid extracted from certain essential oils especially from clove oil) also includes 1 cassette of balsam fragrance, 2 bottles of balsam oil and six jars of balsam against cold [87]. Such records attest to different composition and uses of balsam. High officials explicitly ordered foreigners to obtain specific Western medicines for the court, e.g. the Guangdong grand coordinator and provincial governor (*Guangdong xunfu* 廣東巡撫) Fan Shichong 范時崇 (1663*–*1720).

The Kangxi Emperor 康熙 (r. 1662*–*1722) highly valued certain western medicinals and also asked missionaries to prepare these at court. He also provided his officials with certain of these drugs. Charlotte Furth and Marta E. Hanson even speak of a “medical pluralism” during his reign period [88]. Well-known is the story of how the Jesuits came to cure Kangxi of a malarial fever with so-called Peruvian bark. According to a 1698 account of the French Jesuit Joachim Bouvet, the leader of the French delegation, Father de Fontaney, had successfully used *quinquina* or Peruvian bark to cure the emperor of a malignant fever in July 1693, after the substance had successfully been tested on three men with malarial fever and four healthy members of the imperial clan [89]. Kangxi highly valued the Peruvian bark, and the story definitely demonstrates how promptly a new medicinal drug could be adopted at that time [90]. He is quoted with the words: “The West has a kind of tree bark called *Jinjiqin* that cures illnesses of intermittent fevers (i.e. malaria) with just one dose. Thus, one can see that using medicines is a case of [using them for] the right syndrome” [91].

Kangxi also ordered the French Jesuit Jean-François Gerbillon (1654*–*1707) to translate western knowledge on medicinal drugs into Manchu, and Gerbillon, together with Dominique Parrenin (1665*–*1741), later composed a Manchu handbook of western medicinal drugs and practices, *Xiyang yaoshu* 西洋藥書 (Treatise on Western Medicinals) [92], which is preserved in the Gugong library in Beijing [93].

Although missionary activities were quite restricted under the Yongzheng 雍正 Emperor (r. 1723*–*1735), western medicinal and pharmaceutical activities of the missionaries at the court continued. *Haiguo sishuo* speaks of balsam oil as a gift of Italian and Portuguese missions in 1725, 1727 and 1753 [94]. A document preserved in the First Historical Archive in Beijing offers us a kind of inventory list of a workshop within the palace from 1726. The inventory includes, for example, two glass and three tin bottles of balsam oil, eighteen balsam fragrance balls, two tin bottles of balsam oil originating from Peru (畢錄地方出的巴爾薩木油二錫瓶) and a coconut box of balsam fragrance to prevent a cold. That here a distinction is made between the Peruvian balsam and two bottles “originating from Peru” indicates either that they may have reached the court directly from Spanish America and were traded on the Manila galleons, or, it shows that this bark was also imported from some other regions in the Spanish American or Indian Ocean world (like the quinine that had been brought from India by Claude de Visdelou (1656*–*1737), and with which the Kangxi Emperor had been treated) [95], perhaps of lower quality.

We encounter here another use of the balsam, namely as fragrance balls.

Yongzheng is also quoted with the words: “This oil, this fragrance is stored everywhere in the Hall of Martial Valour and the Hall of Mental Cultivation, it has become known through correspondence with Westerners” (此油、此香武英殿、養心殿盡有收貯的,著西洋人配合) [96].

At court, Peruvian balsam was valued and used against a cold or beginning influenza but, above all, it was very important in curing sword wounds and for external injuries and was, consequently, highly valued among the Manchu military. A Mongol general in the Qing army requested that the court should provide more of this substance:

On the 6^th^ day at the beginning of the 2^nd^ month of 1734 (Yongzheng 24), the Grand Minister Haiwang (?*–*1755) gave the following imperial order: ‘According to what the consort Cering [1672*–*1750] [97] has memorialized, balsam oil is extremely useful in the army; You should take care of (provisioning us) with this oil, the more the better, and store it in tin bottles, which are (normally) used as tea caddies, in order to make it into solid packages, and then take them all to Cering, the emperor’s son-in-law, for his disposal (of it to the army). This was endorsed. On the 9^th^ day of the same month, the manager of the Bureau of Provisions prepared 20 *jin* of balsam oil that was stored in 20 large and 4 small tin bottles as well as in 16 tea tin bottles at the palace workshop, in total more than 40 bottles.

雍正十二年二月初六日內大臣海望奉旨:據額附策淩奏稱,巴爾撒木油軍前深為適用,爾將油多多料理些,用盛茶錫瓶盛裝,務期堅固包裹,帶與額附策淩應用。欽此。於本月初九日司庫常保將巴爾撒木油二十斤盛在造辦處做得大錫瓶二十瓶、小錫瓶四瓶、盛茶錫瓶十六瓶,以上共四十瓶 [98].

Faced with its usefulness as a remedy in the army, it is not surprising that the available Peruvian balsam from Spanish America circulated more widely and was more widely applied during Qing times than the balsam that originated from the Middle East.

We have already mentioned that balsam was also appreciated in the form of fragrance or incense balls, aimed to improve the smell of the air. In 1763, the Qianlong 乾隆 Emperor (r. 1736*–*1795) decreed that the Portuguese missionary Manuel de Mattos (1725*–*1764) provide “balsam fragrance” 巴拉薩嗎香 [99]. A black bottle of balsam oil is still preserved in the Gugong Palace Museum in Beijing. It carries two lines of characters, one saying “diluted balsam oil, net weight 1 catty and 9 *liang* (= approx. 586 g)” (稀巴爾撒末油淨重一斤九兩), the other saying “In the beginning of the 5^th^ month, on the 7^th^ day, 1763, [the imperial physician] Bai Shixiu asked for 8 *qian* (ca. 30 g) of balsam oil to use it for balsam fragrance to prevent colds (乾隆二十八年五月初七日,白世秀討去巴拉薩嗎油八錢,配避風巴拉薩嗎香用) [100].

Such information does not only demonstrate the appreciation of the balsam among ruling elite circles but also attests to a relative strict control of the substance. Especially in face of its use for the curing of wounds in the army, its use against colds and chills and very obviously the appreciation of its fragrant flavour by the social and ruling elites turned balsam into a substance very much in demand, one that was, at the same time, not provisioned in very large quantities. Mostly, it reached the court as official gifts. In the Hall of Martial Valour 武英殿 of the palace in the Forbidden City, there was a so-called Room for the Preparation of Aromatic Medicines and Liquids (*lufang* 露房). This was the place where medicines from the Europeans (lit. the Western Ocean) and medicinal liquids distilled from flowers were stored. In the summer of 1814, an inventory was taken and at that time the store room was filled with lots of bottles, containing the oils of cloves, nutmegs, cinnamon, etc. The oils had mostly already thickened into a kind of paste, as it had been confined to bottles for a long time. In addition, there were all kinds of animal parts and essences, from spiders, dogs, turtles, lions or snakes, including snake eyes. Then there was also a substance called theriac (*deliyake* 德力雅噶; a medical concoction, a kind of elixir, used as an antidote against snake bites and poisoning), that looked like an ointment, etc. Originally, all this was managed by western missionaries, which is why there were so many western medicines [101]. Also Yao Heng 姚衡 (Qing) mentions the “Room for the Preparation of Aromatic Medicines and Liquids”, where Europeans prepared their medicines. In total, there were 220 kinds of oils that had been bestowed as gifts, among all these substances balsam oil (3 *jin*, 1 *liang*, 3 *qian* and 5 *fen*), six glass bottles, able to cure sword and knife injuries (巴爾撒米油三斤一兩三錢五分六玻璃瓶,治刀傷) [102].

Missionaries not only brought medicinal knowledge and drugs from the New World, the Spanish colonies in America, via Europe or the Pacific and Manila to China and Japan, but also sent prepared medicines to Manila. As Cui Weixiao 崔維孝 has shown, due to the activities of Blasius García (1635*–*1699), Franciscan physicians annually sent to Manila great quantities of medicinals and aromatics, such as theriac (*jiedu yao* 解毒藥), *Hyacinthus orientalis* (*fengxinzi* 風信子), brown sugar (*hongtang* 紅糖, *Saccharon Granulatum* Rubrum), rhubarb pills and refined rhubarb, *bálsamo apoplético* (中風香脂藥膏) and others. The hospital in Guangzhou, vice versa, received from Manila substances such as jalap root resin (球根牽牛根莖), *Salvia japonica* Thunb. (鼠尾草), *carthamus* flower (紅花, *Carthami Flos*), *bálsamo* (香脂藥膏), *Pini Resina* essence (松脂精), and *Tamarindus indica* (羅望子果) [103]. The balsams mentioned here are not identical with the balsam of Peru. Most of these substances were probably shipped via Manila to China across the Indian Ocean, but, as ship records about cargoes and archaeological evidence show, other medicinal drugs were without question shipped across the Pacific.

After the Kangxi and Yongzheng reign periods, many of these western medicinals were apparently more and more seldomly used. Especially after the Qianlong reign, much of the former knowledge was lost, so that later in the nineteenth century many scholars were no longer acquainted with such substances, their uses and applications and their origins (see also below).

That interest in the embalming of corpses in Ming times obviously weighed higher than the use of balsam for medicinal purposes is also reflected by *Wuli xiaoshi*. Fang Yizhi includes a separate paragraph entitled “method for preserving corpses” (*liushi fa* 留屍法).

### Method of preserving corpses

“When Li Yu [104] ingested jade [105], his corpse did not stink in the summer; when the orifices are sealed with gold, it also does not decay. When pouring mercury, this is still more efficient, or soaking it with tea through the nose, then you can also prevent stinking during the summer. *Hanshu* says: The foul and despicable corpse of Empress Lü (?*–*180 BCE) was restrained with quicksilver. In Peru, there is a balsam tree that produces a resin to embalm corpses so that they do not decompose even after 1000 years. The *Quebian lu* says: when a disciple gives away his master’s [dead corpse] because he died in a sitting posture [106], *nao* mineral is mixed with Borneo camphor, and together with dry mercury put into the nose holes and the body is vapoured with medicinal aromatics. If one wants to pass away with the vapours peacefully and quietly exhausting, with the head not feeling warm, they must escape the dead body from the head. After a long time, [the corpse] will dry by itself; this is the special way of Theravada [Buddhism, lit. “the small vehicle”] to reach the status of [unification with nature]. Is this not sufficient to startle? Bones and hard matter do not decay, the will congeals and the essence is bound to become a demon. If one burns what is not evil, this is the origin of the Buddhist Śarīra relic. What is said in the explorations of thing is that hair and organ of a boy can be stuck to the Śarīra relic, when frankincense remains long, it can bear a Śarīra.”

留屍法 李預服玉其尸暑中不殠黄金塞竅亦不腐灌澒更效或以茶灂澒納鼻亦可暑中不殠漢書言汙辱呂雉尸以水銀斂也孛露國有一拔爾撒摩樹脂塗尸千年不朽確辨録曰有徒以坐脱販其師者合婆律也乾澒納鼻而耨香熏為肉身者也寡欲恬澹气盡而逝心不係戀煖從頂脱尸為遺蜕久之自乾小乘專致常然豈足怪乎骨剛者不壞志凝精結有成塊燒不壞者此舍利子之所由來也物理所曰童男髪根可黏起舍利乳香久留能生舍利.

Why Fang Yizhi was so interested especially in the embalming of dead corpses in ancient Peru is an interesting question. We know from ancient, especially Han and pre-Han period, tombs that members of the ruling and social elites in China also frequently embalmed their dead. Lady Dai from the Mawangdui 馬王堆 tomb complex (168 BCE) is perhaps one of the most well-known examples. Well preserved mummies were also discovered from the Ming and Qing dynasties [107]. The practice was, consequently, still known during Fang Yizhi’s time. He was obviously quite impressed by the fact that Andean people also possessed an ancient technique of preserving dead bodies. That information about the knowledge of mummification in a country overseas was more interesting and fascinating to scholarly elites in contemporary China is also attested to in the maps we have introduced above, which provide this information regarding their Andean “islands in the sea”. Medicinal purposes, in contrast, were at least less intriguing.

The Spanish, too, were excited about the mummification practices of the Incas and the Andean people in general. In 1559, they confiscated and displayed the best-preserved mummies in Lima’s most sophisticated centre of European healing and botanical knowledge, the Hospital of San Andrés [108]. Possibly, the knowledge was used to mummify notable deceased persons in the Spanish Philippines. There is one entry in the Archivo General de Indias (AGI) where a surgeon, Joaquín de Castro (*cirujano*), requested the authorities to pay for the medicines and the labour to embalm the cadaver of the archbishop of the Philippines (1729–1732), Carlos Bermúdez de Castro (1678–1729) [109]. Obviously, also animals were embalmed, it seems even on board of ships, as an entry from the AGI dated 1778 suggest: Don Juan Francisco de Anda y Salazar [“oydor de la Audiencia de Manila and executor (*albacea*) of Simón de Anda y Salazar” (1709–1776), governor of the Philippines from July, 1770 to October 30, 1776)] took care of a cargo of animals for the Spanish King, among them one elephant, four small deer from Batavia, two deer from Coromandel and a dove from Ternate; accordingly, the elephant and three of the four deer reached their destination alive and the others embalmed (“y los demas embalsamados”) [110].

Other entries by Fang Yizhi are directly referred to *Zhifang waiji*, for example a note stating that the Indians of the Peruvian Andes Mountains have a fountain of a liquid like grease that the local people extract to burn or use it as oil paint. All oil fires using oil from these wells burn even better when sprinkled with water, and are extinguished only when dabbed with earth dust (外紀寡斯大山孛露有泉如脂膏人取燃燈或作油漆用凡井油火以水澆之愈熾以地灰撲之則滅) [111].

#### Japan

**Fig. 5:**
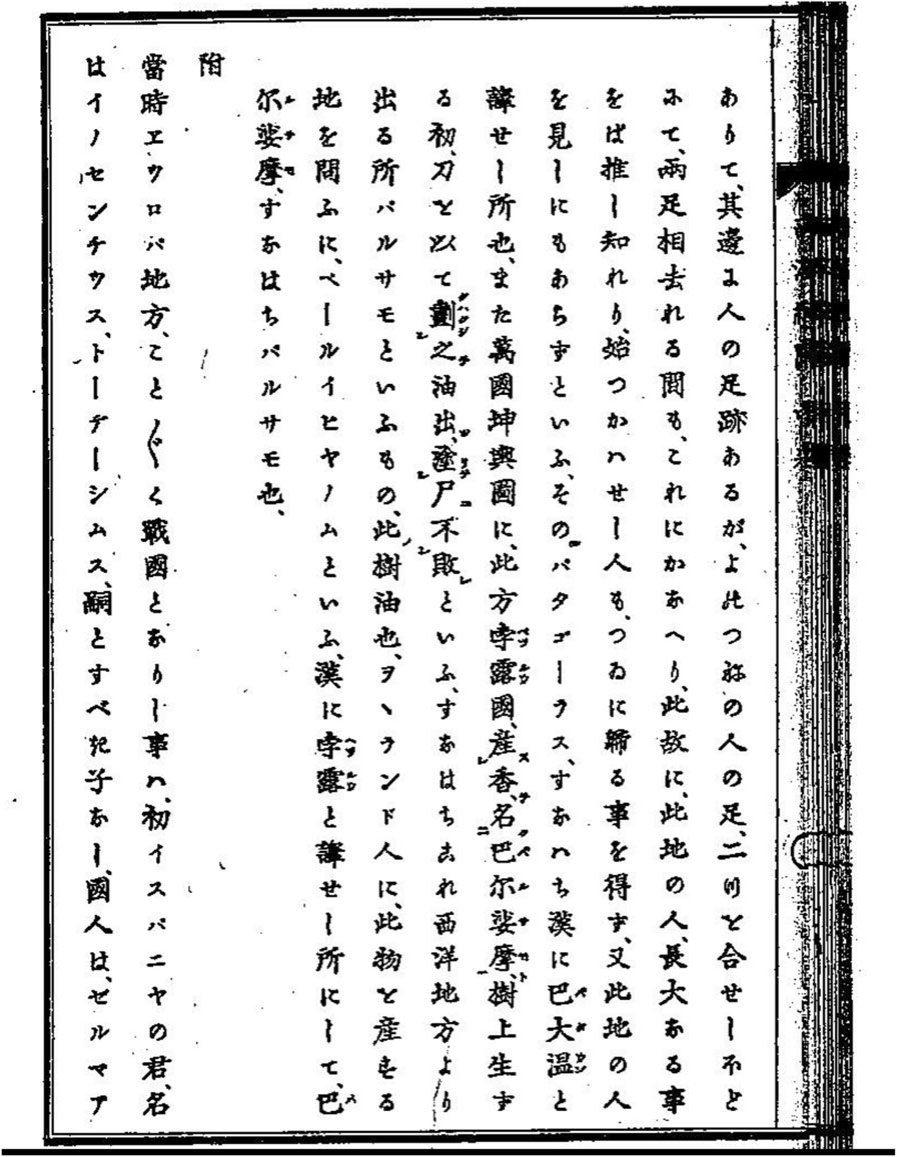
Entry on balsam in Keiyō kibun 西洋紀聞 (p. 18b)

**Fig. 6:**
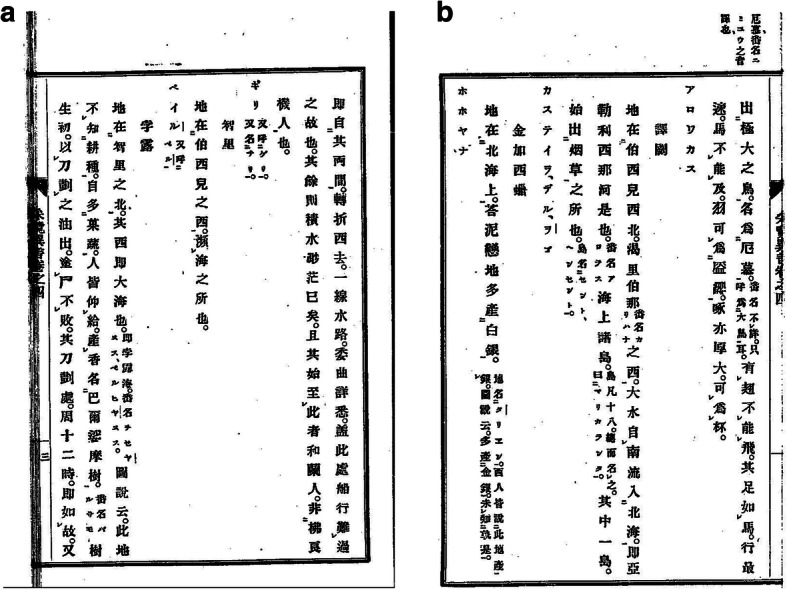
**a** and **b** Entries on balsam in Sairan igen 采覽異言

The reception of western medicine was different in Japan, where through Dutch medicinal knowledge a separate medicinal school evolved, “Rangaku” 蘭學 (Dutch medicinal learning). Generally speaking, we can observe that Japanese scholars were more open towards new knowledge and practices from Europeans. The world map of Matteo Ricci, *Kunyu Wanguo quantu* 坤輿萬國全, had a great impact on local scholars, after it became known in Japan. In 1708, Arai Hakuseki 新井白石 (1657–1725), a well-known Neo-Confucian scholar, official and writer in the Japan of that time, produced a painted, coloured version of the map *Kunyu Wanguo quantu*. He also copied Ricci’s entry on balsam 巴尔娑摩, in his map written as 巴尔婆摩. In 1713, he wrote a three-volume treatise on the Occident entitled *Keiyō kibun* 西洋紀聞 (Chronicle of the West), a work that was mainly based on his conversations with the Italian missionary Giovanni Battista Sidotti (1668–1714). The second volume is a description of the five continents, that is, Africa, Asia, Australia, Europe and the Americas. There, Hakuseki translates the information from Ricci’s map into Japanese, and explains that in Spanish America there is a country called Perukoku 孛露國, where a fragrance is produced called 巴尔娑摩. The tree, when cut with a knife, produces an oil which is used to embalm dead corpses so that they do not decompose. The balsam (バルサモ) that comes from the western countries (i.e. that is traded by the Europeans) is exactly the oil of this tree. I asked some Dutch people about the location of this product and they replied it was Peru. In Chinese, this is translated into Polu 孛露. *Ba’ershamo* 巴尔娑摩, this is balsam (バルサモ) (see Fig. 5) [112]. In his *Sairan igen* 采覽異言 (Strange Stories Acquired), he almost literally copies from Ricci, because, in contrast to his explanations in *Keiyō kibun*, he uses mainly Kanji 漢字. Interestingly, he also adds geographical information, stating that Peru lies in the north of Chile, lying adjacent to a large ocean in the west. And he introduces emus, large birds that cannot fly and have feet resembling horses (see Fig. 6a, b) [113].

All this demonstrates that Arai Hakuseki intensively engaged with the knowledge about these foreign countries and their products. In *Keiyō kibun*, he explicitly states that he discussed the location of Peru with Dutch people. Dutch medicine applied various balsams in its treatments. So Dutch physicians offered another channel to obtain more knowledge on the uses of balsam as a medicinal.

**Fig. 7:**
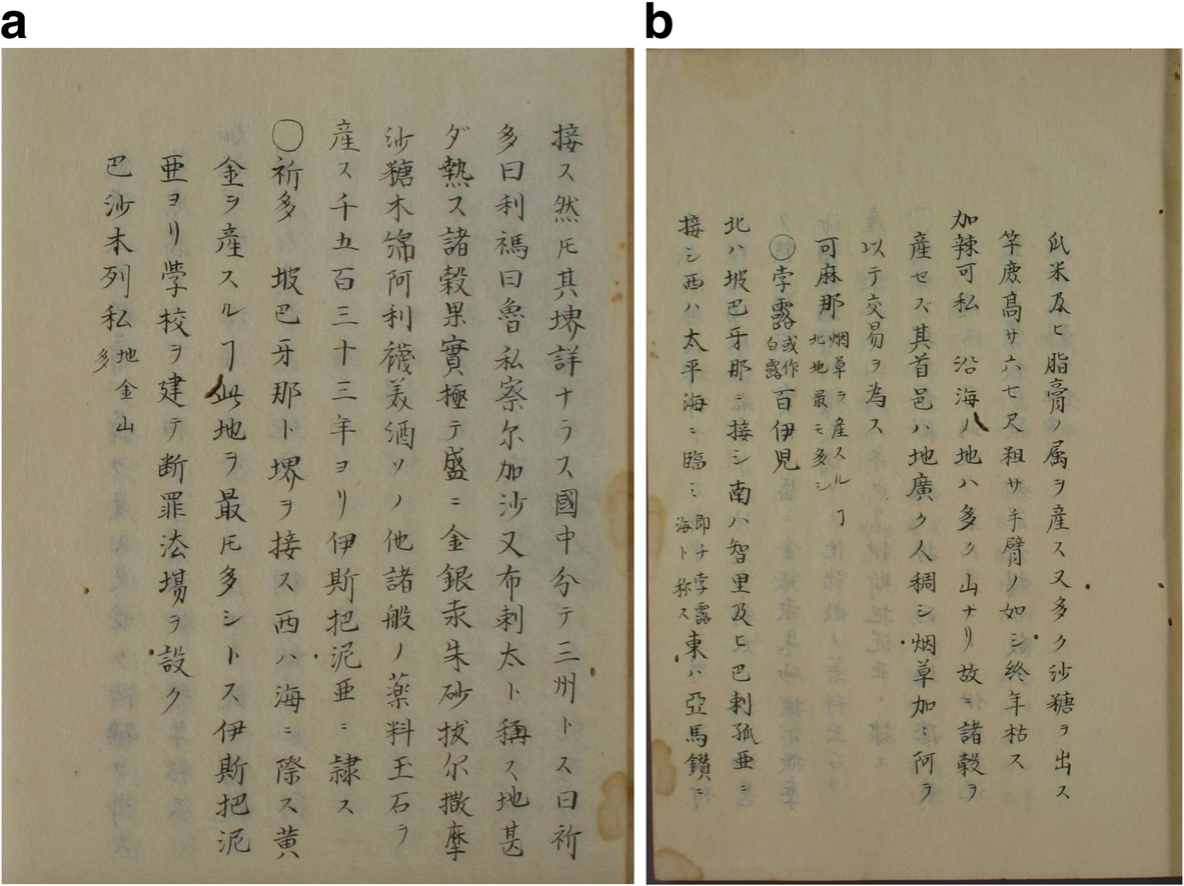
**a** and **b** Entries on balsam in Hon‘yaku chikyū zenzu ryakusetsu 翻譯地球全圖略說

Katsuragawa Hoshū 桂川甫周 (1751–1809), a Japanese physician and Rangaku scholar who served the Tokugawa Shogunate 徳川幕府 (1603–1868) as a physician and translator of Dutch, translated the world map by Willem Janszoon Blaeu (1571–1638) into Japanese, *Hon‘yaku Chikyū zenzu ryakusetsu* 翻譯地球全圖略說 (Translation and Brief Account of the Entire World Map) in 4 *juan* [114]. He also mentions balsam (拔尔撒摩) as a product of Peru, together with gold, silver, mercury, cinnabar (朱砂), sugar (沙塘), cotton, olive oil (阿利襪美油) [115] and numerous kinds of medicinals, jade and precious stone, that are locally produced and since 1533 belong to Spain. He was even aware of the Spanish administrative-bureaucratic division, and the “audiencias” in their viceroyalties in the Americas, and mentions here Quito 祁多, Lima 利禡 and Juríes 魯私 (?) in Charcas 察尔加沙 (see Fig. 7a, b) [116].

**Fig. 8:**
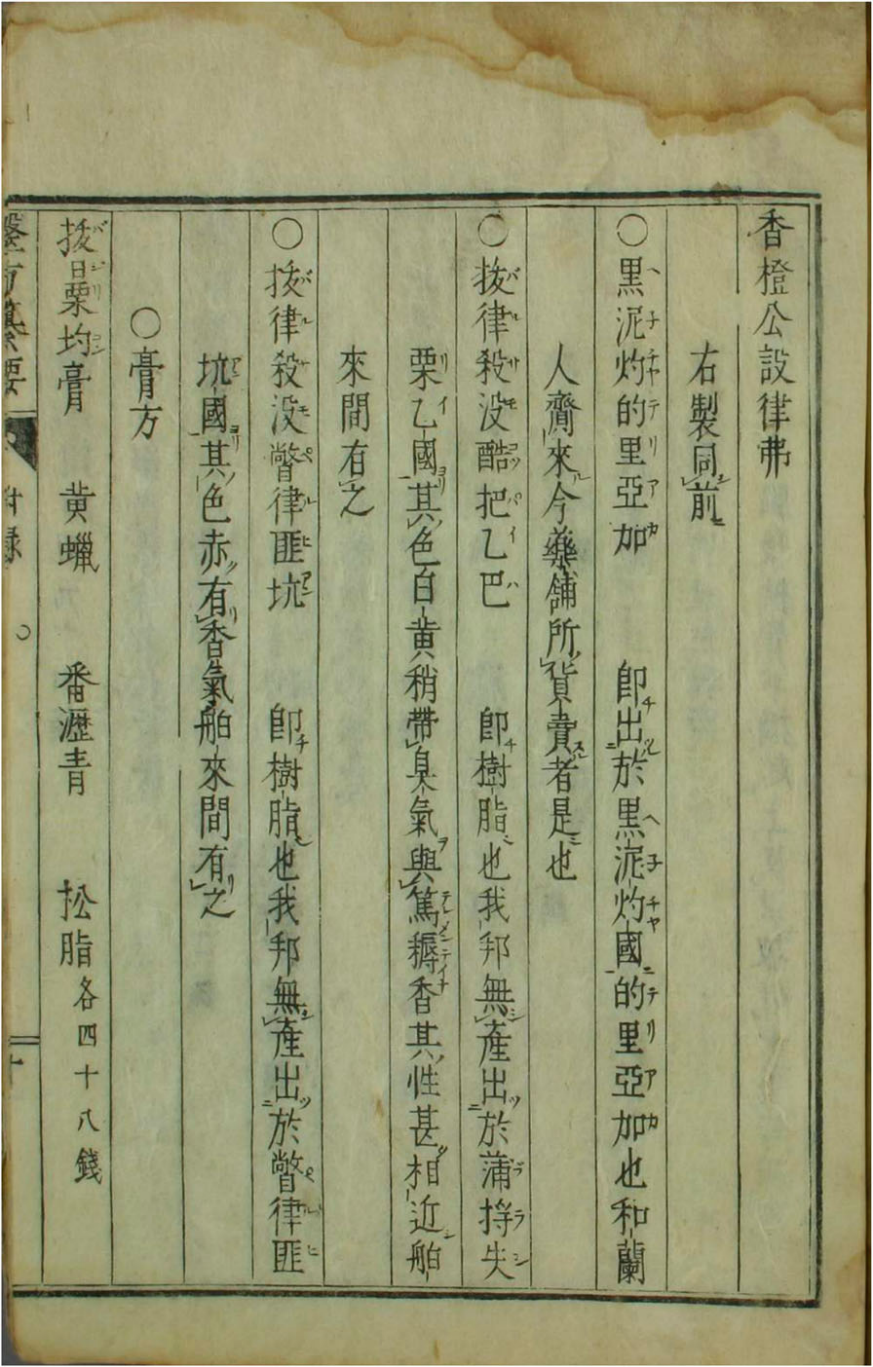
Entry on balsam in Oranda ihō san’yō和蘭醫方纂要 (4 fulu, p. 10a)

In 1817, Ema Shōsai 江馬松斎 (Genkō 元弘, 1779–1820) translated and printed the *Oranda ihō san’yō* 和蘭醫方纂要 (Compendium of Dutch Medicinal Recipes) [117]. He also mentions various kinds of balsam. Especially interesting is the information about a Peruvian balsam tree that produces an oil, is reddish in colour and fragrant. It is imported by ships (拔律殺沒瞥律匪坑:即樹脂也,我邦無產,出於瞥律匪坑國。 其色赤,有香氣。舶來間有之) (see Fig. 8) [118].

**Fig. 9:**
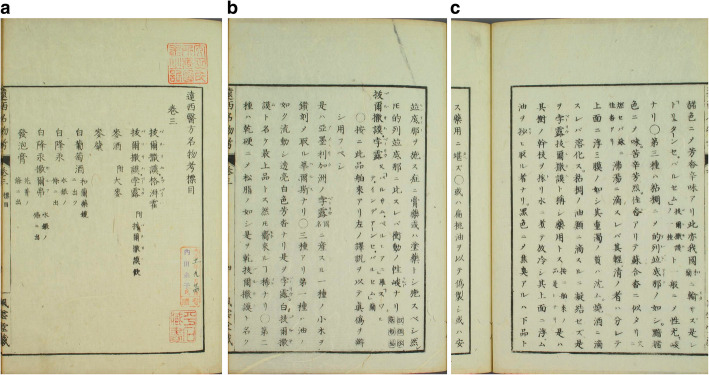
**a**, **b**, and **c** Entries on balsam in Ensei ihō meibutsukō 遠西醫方名物考

Yoshio Shunzō 吉雄俊藏 (1787–1843) translated a manual written for physicians on board of ships by the seventeenth-century Dutch official surgeon, Jan Kouwenburg, *Shinyaku Oranda neikai yōhō* 新譯和蘭內外要方 (New Translation of the Essential Methods of Dutch Internal Medicine and Surgery; 1820) that also explicitly refers to balsam of Peru (拔爾撒謨百露比亞泥) [119]. In the nineteenth century, in contrast to China, interest continued unabated. In the 1820s, Udagawa Genshin 宇田川玄真 (1769–1834) and Udagawa Yōan 宇田川榕菴 (1798–1846) [120] translated and annotated *Ensei ihō meibutsukō* 遠西醫方名物考 (A Study of Far-Western Medicinal Recipes and Notable Things) that provides even more information on the Peruvian balsam: “Bo’ersamo Poli, also called *barusamu peruviana* in Latin, and *zuid indiaanse balsam* in Dutch” (拔爾撒謨孛露 又「バルサム.ペルビアニ」羅「スワテ.インデアーンセ.バルセム」蘭). They describe the tree and also its medicinal uses and indications: it strengthens the nerves, prevents decomposition, can be used as a laxative, with cleaning functions, to sweat out excretions, can dissipate cold tuberculosis and is also effective against pneumoconiosis, asthma, gonorrhoea and other deficiency diseases [121]. This is definitely the most detailed and also the most up-to-date description about the balsam of Peru (see Fig. 9a–c). In the later nineteenth century, one continues to encounter relatively frequent references to the Peruvian balsam and its medicinal properties and uses in Japanese medical literature—quite in contrast to China, where the knowledge that had existed during the Ming, early and high Qing seems to have been almost lost, as the following example may show.

Yu Yue 俞樾 (1821–1907) was a very prominent Qing scholar, specialized in philology and textual studies, including history. In his scholarly notes, *Chaxiang shi congchao* 茶香室叢鈔, he copies Fang Yizhi’s *Wuli xiaoshi* and adds his remarks: “I do not know where the country of Peru is located, and also do not know what kind of tree this balsam is” (孛露國不知何地,拔爾撤摩亦不知何樹) [122]. That an erudite intellectual in the late nineteenth century had absolutely no idea about the whereabouts of a country like Peru seems hardly credible. It demonstrates that Yu Yue was not sufficiently interested in the world beyond, nor in the knowledge that had been collected by scholars over the last centuries. As we have seen above, another late Qing scholar, Wei Yuan, simply copied the information from earlier works. There was no further “digestion” or development of the knowledge on the balsam of Peru in late Qing scholarship. The interest was pure encyclopaedic and philosophic.

## Conclusions

What conclusions can we draw regarding the transfer of knowledge related to the use and application of Peruvian balsam?

Knowledge about the substance was brought to China through European missionaries. Initially, balsam was probably only used in the hospitals and pharmacies of European missionaries. But the knowledge gradually reached the court and the social and ruling elites of China. Especially the Kangxi Emperor possessed a very positive attitude towards Western medicine, and even wished to have European physicians and pharmacists at court. He definitely sponsored the dissemination of Western medicine in China. The French Jesuits Joachim Bouvet (1656*–*1730) and Jean-François Gerbillon (1654*–*1707) even set up a pharmaceutical laboratory with all the necessary equipment at the request of Kangxi [123]. The general commercialisation of medicine in Ming and Qing China quickly had so-called “new” drugs enter into contemporary pharmacies and pharmacopoeia, and social and ruling elites profiting from new ideas. We know that the Qing court, for example, basically received its provisions of Peruvian balsam through the networks of missionaries, via the trans-Pacific Manila galleon trade. But if there were special merchants and merchant networks engaged in its provision and cross-oceanic distribution remains a question to be investigated in more detail.

Textual and even archaeological evidence attest to the use of the balsam among the Qing elites and, as we have seen, during the high Qing, also in the Qing army (“balsam oil is extremely useful in the army”), as it was very effective in curing external knife, sword, arrow and other injuries as well as skin diseases, including smallpox [124]. This is a clear and interesting indication of its use beyond the narrow elite circles. But how and when exactly the oil was used in military circles, this is a question that definitely requires further investigation. And textual evidence one way or the other is scanty.

After the Qianlong era, however, the “new” knowledge gradually was lost—in a complete disparity with Japan where the knowledge was continuously updated. Living in an environment where contacts to the outside world were the exception rather than the rule, many Japanese physicians were eager to learn more about medical practices from abroad. Especially through the mediation of Dutch physicians and their medicinal knowledge—then among the most advanced in the early modern world—Japanese scholars, doctors and pharmacists came to have a solid knowledge about the balsam of Peru, its origin and its medicinal uses. By contrast, most Chinese physicians, medical theorists and intellectuals were preoccupied with metaphysical and cosmological interpretations of their own classical traditions, seeking a “return to antiquity” (*fugu* 復古). After the Opium Wars (1839–1842 and 1856–1860), many also felt threatened by the West and by Western traditions. In this context, starting in the second half of the nineteenth century, the idea of using “Chinese learning as substance, and Western learning for practical use” (*Zhongxue wei ti, Xixue wei yong* 中學為體, 西學為用) became very widespread and popular among Chinese thinkers. This may explain, at least partly, why their interests in Western knowledge were strictly limited. If such knowledge was not directly related to Western technology and military systems that could be used to strengthen the country (for “practical use”, in other words), it remained basically encyclopaedic and philosophic.

## Data Availability

Sources, texts and maps, are either available as printed books and source collections or through open access archives of libraries, all hyperlinks have also been included in the references. Wiki text project: https://ctext.org/wiki.pl?if=en&chapter=443948&remap=gb https://ctext.org/wiki.pl?if=gb&res=520749 https://ctext.org/wiki.pl?if=gb&res=259380 https://ctext.org/wiki.pl?if=gb&res=929696 https://ctext.org/wiki.pl?if=gb&res=87006 https://ctext.org/wiki.pl?if=gb&chapter=566144 https://ctext.org/wiki.pl?if=gb&res=99548 https://ctext.org/library.pl?if=gb&res=3650 https://ctext.org/wiki.pl?if=gb&res=722035 https://ctext.org/wiki.pl?if=gb&res=7 https://ctext.org/wiki.pl?if=gb&res=268470 https://ctext.org/wiki.pl?if=gb&res=371623 https://ctext.org/library.pl?if=gb&res=81155 https://ctext.org/wiki.pl?if=gb&res=303463 https://ctext.org/library.pl?if=gb&res=3276 National Library of Australia: https://nla.gov.au/nla.obj-48294144/view?partId=nla.obj-48294350#page/n2/mode/1up US Archives: https://ia800608.us.archive.org/32/items/A050111/A050111.pdf *Frontiers of History in China:* doi:10.3868/s020-004-015-0002-0 National Diet Library, Japan, Digital Collection: https://dl.ndl.go.jp/ Waseda University Library Archives: https://archive.waseda.jp/archive/database.html?arg={}&lang=en https://www.waseda.jp/library/en/collections/special-collections/ https://www.wul.waseda.ac.jp/kotenseki/index_en.html Wiley Online Library: 10.1002/9781444338386.wbeah21264 https://onlinelibrary.wiley.com/doi/epdf/10.1111/cod.13263 http://lawdata.com.tw/tw/detail.aspx?no=279620 https://journals.equinoxpub.com/JMA 10.5962/bhl.title.3538 https://brill.com/view/serial/CLIOON https://catalog.hathitrust.org/Record/008296820 http://www.historicas.unam.mx/publicaciones/publicadigital/libros/caminosymercados/cm029.pdf https://www.ncbi.nlm.nih.gov/pmc/articles/PMC4304538/ 10.5962/bhl.title.98775 John Carter Brown Library, https://archive.org/details/balsamumverumquo00slev https://academic.oup.com/shm/article-abstract/28/1/22/1653726 https://www.zoharamar.org.il/wp-content/uploads/%D7%90%D7%A4%D7%A8%D7%A1%D7%9E%D7%95%D7%9F-%D7%94%D7%A4%D7%A7%D7%94-%D7%A1%D7%A4%D7%A8-%D7%93%D7%A0%D7%99%D7%90%D7%9C.pdf https://www.kanripo.org/ed/KR3j0160/WYG/003#1a, 003-29a; https://www.kanripo.org/text/KR3j0160/009, 009-5b; https://ctext.org/wiki.pl?if=gb&chapter=160695#p31; https://sou-yun.cn/eBookIndex.aspx?kanripoId=KR2m0013_097&id=278, 97.23a; https://www.kanripo.org/text/KR2k0009/199, 199-54a; https://www.academia.edu/29921776/Evaluation_of_Latin_America_Materia_Medica_and_is_influence_in_therapeutic.pdf https://www.brown.edu/Facilities/John_Carter_Brown_Library/exhibitions/drugs/pages/first.html http://bibliotecadigital.aecid.es/bibliodig/es/consulta/registro.cmd?id=579 https://openaccess.leidenuniv.nl file 131483.pdf Archivos españoles, https://pares.mcu.es https://ojs.lib.umassd.edu/index.php/plcs/article/view/PLCS17_18_Walker_page77 https://wellcomecollection.org/works/uxa7cnzk https://biblio.ugent.be/publication/446686 http://connection.ebscohost.com/c/articles/85295634/shenjianlu-by-fan-shou-yi-reconsidered-new-geo-historical-notes https://www.ncbi.nlm.nih.gov/pmc/articles/PMC6158963/ https://babel.hathitrust.org/cgi/pt?id=ucm.5329198652&view=1up&seq=5 10.3998/saksaha.13401746.0008.001 Further links: Drugbank https://www.drugbank.ca/drugs/DB11482 The Recipies Project: https://recipes.hypotheses.org/3107 https://recipes.hypotheses.org/8092

## Abbreviation

AGI: Archivo General de Indias, Sevilla, Spain
Xuxiu: Xuxiu Siku quanshu 續修四庫全書, Xuxiu Siku quanshu bianzuan weiyuanhui 續修四庫全書編纂委員會, ed. (Shanghai: Shanghai guji chubanshe, 1995)
